# Radiotherapy plus Concomitant Adjuvant Temozolomide for Glioblastoma: Japanese Mono-Institutional Results

**DOI:** 10.1371/journal.pone.0078943

**Published:** 2013-11-12

**Authors:** Takahiro Oike, Yoshiyuki Suzuki, Ken-ichi Sugawara, Katsuyuki Shirai, Shin-ei Noda, Tomoaki Tamaki, Masaya Nagaishi, Hideaki Yokoo, Yoichi Nakazato, Takashi Nakano

**Affiliations:** 1 Department of Radiation Oncology, Gunma University Graduate School of Medicine, Maebashi, Gunma, Japan; 2 Department of Neurosurgery, Gunma University Graduate School of Medicine, Maebashi, Gunma, Japan; 3 Department of Human Pathology, Gunma University Graduate School of Medicine, Maebashi, Gunma, Japan; University of Michigan School of Medicine, United States of America

## Abstract

This study was conducted to investigate the feasibility and survival benefits of combined treatment with radiotherapy and temozolomide (TMZ), which has been covered by the national health insurance in Japanese patients with glioblastoma since September 2006. Between September 2006 and December 2011, 47 patients with newly diagnosed and histologically confirmed glioblastoma received radiotherapy for 60 Gy in 30 fractions. Among them, 45 patients (TMZ group) received concomitant TMZ (75 mg/m^2^/day, every day) and adjuvant TMZ (200 mg/m^2^/day, 5 days during each 28-days). All 36 of the glioblastoma patients receiving radiotherapy between January 1988 and August 2006 were analyzed as historical controls (control group). All patients were followed for at least 1 year or until they died. The median survival was 15.8 months in the TMZ group and 12.0 months in the control group after a median follow-up of 14.0 months. The hazard ratio for death in the TMZ group relative to the control group was 0.52 (P<0.01); the 2-year survival rate was 27.7% in the TMZ group and 14.6% in the control group. Hematologic toxicity of grade 3 and higher was observed in 20.4% in the TMZ group. Multivariate analysis showed that extent of surgery had the strongest impact on survival (P<0.01), while the use of TMZ had the second largest impact on survival (P = 0.035). The results indicate that combined treatment with radiotherapy and TMZ has a significant survival benefit for Japanese patients with newly diagnosed glioblastoma with slightly higher toxicities than previously reported.

## Introduction

Glioblastoma is the most aggressive type of primary brain tumor and accounts for approximately 52% of all primary brain tumor cases [1]. Regardless of advances in microsurgery techniques, radiotherapy (RT) and chemotherapy, the survival rate for glioblastoma has remained very low, and most patients with glioblastoma die within 2 years [2]. Standard therapy for glioblastoma consists of maximal surgical resection within safe limits, followed by RT. Chemotherapeutic agents, including nitrosourea, have been used concurrently with RT and/or in an adjuvant setting [3]. However, the addition of chemotherapeutic agents to RT resulted in limited success for survival [4]. A meta-analysis based on 12 randomized trials showed a small survival benefit from the combined use of chemotherapy and RT compared with RT alone. In the meta-analysis, a 5% increase in survival at 2 years was observed in patients treated with the combined therapy. However, the meta-analysis included 37% of patients with prognostically more favorable lower-grade gliomas [4].

Temozolomide (TMZ, marketed as Temodar by Schering-Plough, USA) is an oral alkylating agent. TMZ has been shown to improve the survival of patients with glioblastoma in concomitant and adjuvant use with RT. In 2005, a randomized phase III trial by the European Organization for Research and Treatment of Cancer (EORTC) and the National Cancer Institute of Canada Clinical Trials Group CE3 (NCIC), including 573 glioblastoma patients, established TMZ as the standard chemotherapeutic agent for glioblastoma treatment [5]. In their study, after surgical resection, TMZ (75 mg/m^2^/day×7 days/week for 6 weeks) was administered concomitantly with RT (total dose of 60 Gy delivered by a schedule of 2 Gy/day×5 days/week for 6 weeks) and in the adjuvant setting (200 mg/m^2^/day×5 days, every 28 days for 6 cycles). After adding TMZ to RT, the median survival improved from 12.1 to 14.6 months, and the 2-year survival rates improved from 10.4% to 26.5%. The long-term results of the trial published in 2009 demonstrated that the 5-year survival rates of the patients treated with the combined therapy and those receiving RT alone were 9.8% and 1.9%, respectively. These results further confirmed the efficacy of adding TMZ to RT [6].

Accordingly, TMZ treatment for high-grade gliomas has been covered by health insurance in Japan since September 2006. However, the survival benefit from the combined treatment with RT and TMZ in Japanese patients with glioblastoma has not been reported. Thus, in this study, patients with newly diagnosed glioblastoma treated with RT plus TMZ in Gunma University Hospital were retrospectively investigated. The survival of TMZ-treated patients and our institutional historical data of glioblastoma patients treated with RT ± various chemotherapeutic agents were compared.

## Materials and Methods

### Ethics Statement

Anonymity of the patients was preserved. All patients provided their written informed consent to participate in this study. The current study was approved by the Gunma University Hospital, Gunma, Japan. The institutional ethics committee exempted the current study from the usual review process because of its retrospective nature.

### Patients

Between September 2006 and December 2011, a total of 47 patients were newly diagnosed with histologically confirmed glioblastoma (World Health Organization [WHO] grade IV astrocytoma) and received RT at Gunma University Hospital. Among these patients, 45 (95.7%) were treated with a combination of RT and TMZ and included in the present study as the TMZ group, while only two patients were not treated with the combined therapy because of their unfavorable general condition. A series of 36 patients treated with RT ± various chemotherapeutic agents between January 1988 and August 2006 (control group) was utilized as a historical control. All patients were followed for more than 1 year or until they died. Informed consent for the treatment and research was appropriately obtained from all patients, and the anonymity of the patients was preserved. The current study was approved by the Gunma University Hospital, Gunma, Japan. The institutional ethical committee exempted the current study from the usual review process because of its retrospective nature.

### Surgical Resection

Surgical resection of the tumors to the maximal extent within safe limits was performed in all patients. The extent of the resection was classified as gross total resection (GTR), subtotal resection (SR), partial resection (PR) or biopsy.

### Radiotherapy

RT was given with 10 MV X-rays delivered by a linear accelerator. Patients in the TMZ group were irradiated with a total dose of 60 Gy using conventional 3D conformal RT (3DCRT) (2 Gy/fraction; 5 fractions/day; 5 days/week). The radiation field covered the target volume plus 2-cm margins. In the first 50 Gy, the target volume included all of the high-intensity areas on T2-weighted images from magnetic resonance imaging (MRI). In the following 10 Gy, the target volume included all of the gadolinium-enhanced areas on the MRI T1-weighted images.

Patients in the control group were treated using either the conventional, hyper-fractionated (e.g., 1 Gy/fraction; 2 fractions/day; 5 days/week) or hypo-fractionated (e.g., 2.5 Gy/fraction; 2 fractions/day; 2 days/week) regimen. To compare the total doses delivered by different regimens, the biological effective dose (BED: α/β ratio = 10.0) was used as previously described (e.g., 60 Gy/30 fractions = BED of 72 Gy) [1].

### Chemotherapy

Patients in the TMZ group received TMZ (75 mg/m^2^/day) every day during the RT period. After a 4-week break, the patients received adjuvant TMZ (200 mg/m^2^/day) according to the standard 5-day schedule every 28 days as long as it was tolerable. Among the 36 patients in the control group, 20 patients received chemotherapy with the PAV (procarbazine, nimustine and vincristine) regimen or other regimens. Nine patients received RT alone. Information on chemotherapy was lost in 7 patients. Interferon-β (IFN-β) was administered in 10 and 7 patients in the TMZ and the control groups, respectively.

### Immunohistochemical Studies

Immunohistochemical studies on O^6^-methylguanine-DNA-methyltranferase (MGMT) and MIB-1 were carried out in the TMZ group. Surgical specimens from the patients in the TMZ group were fixed in neutral formalin immediately after the tumors were resected and embedded in paraffin. The paraffin-embedded tissue sections (5 µm thick) were stained with hematoxylin-eosin. Immunohistochemical staining was performed as previously described [7]. Tumors containing cells stained for MGMT in more than 23.0% of their nuclei were considered positive for MGMT expression, according to a previous study [8]. The positivity for MGMT expression was determined by two pathologists (M. N. and H. Y.). MIB-1 labeling index (LI) was measured using the computer software GunmaLI as previously described [9].

### Statistical Analysis

Continuous variables were analyzed by the Student’s *t* test or Welch’s test in cases in which the variances of the two groups were or were not equal, respectively. Categorical variables were analyzed by Fisher’s exact test. The influence of potential prognostic factors was examined by univariate and multivariate analyses using the Cox proportional hazards model. These analyses were completed with StatMateIII ver. 3.17 (ATMS, Tokyo, Japan). Survival analyses were performed according to the Kaplan-Meier method with two-sided log-rank statistics using JMP 8 (SAS Institute Inc., Tokyo, Japan). Differences were considered statistically significant at P values<0.05.

## Results

The patient characteristics are summarized in **Table 1**. There were no significant differences in patient characteristics, including age, sex, extent of surgery, total radiation dose evaluated by BED and the number of patients receiving IFN-β therapy, between the two groups. Thirty patients (66.6%) in the TMZ group received concurrent treatment with RT and TMZ as planned. Four patients (8.8%) did not receive RT as planned because of disease progression. Ten patients (22.2%) experienced discontinuance or dose reduction of the concomitant TMZ because of hematological toxicities and other reasons. Thirty-nine patients in the TMZ group (86.6%) started adjuvant TMZ and received a median of 7 cycles (range, 0 to 54). The main reason for discontinuing adjuvant TMZ therapy was disease progression. Twenty-one cases (46.6%) in the TMZ group were positive for MGMT expression, and the median value of MIB-1 LI in the TMZ group was 23.0% (range, 5.1 to 65.0%). MGMT expression and MIB-1 LI could not be assessed in one patient because of insufficient paraffin-embedded tissue.

**Table 1:** Characteristics of patients treated with radiotherapy plus temozolomide.

Characteristics	CTR (n = 36)	TMZ (n = 45)	P-value
Age (years)			
Median (range)	59 (9–79)	60 (5–79)	0.71
Sex			
Male	23	29	1.00
Female	13	16	
Extent of surgery			
GTR	11	7	0.26
SR+PR	23	34	
Biopsy	2	4	
Radiation dose (BED; Gy)			
Median (range)	72 (9.6–80)	72 (65–72)	0.30
Adjuvant TMZ (cycle)			
Median (range)		7 (0–54)	–
IFN-β therapy			
Yes	10	7	0.27
No	26	38	
MGMT expression			
Positive	–	21	–
Negative	–	23	
Not assessed	–	1	
MIB-1 labeling index (%)			
Median (range)	–	23.0 (5.1–65.0)	–

CTR, control group; TMZ, temozolomide group; GTR, gross total removal; SR, subtotal removal; PR, partial removal; MGMT, O6-methylguanine DNA metyltransferase; BED, biological effective dose; SD, standard deviation; IFN-β, interferon-β. MIB-1 labeling index could not be assessed in one patient because of insufficient paraffin-embedded tissue.

**Figure 1:**
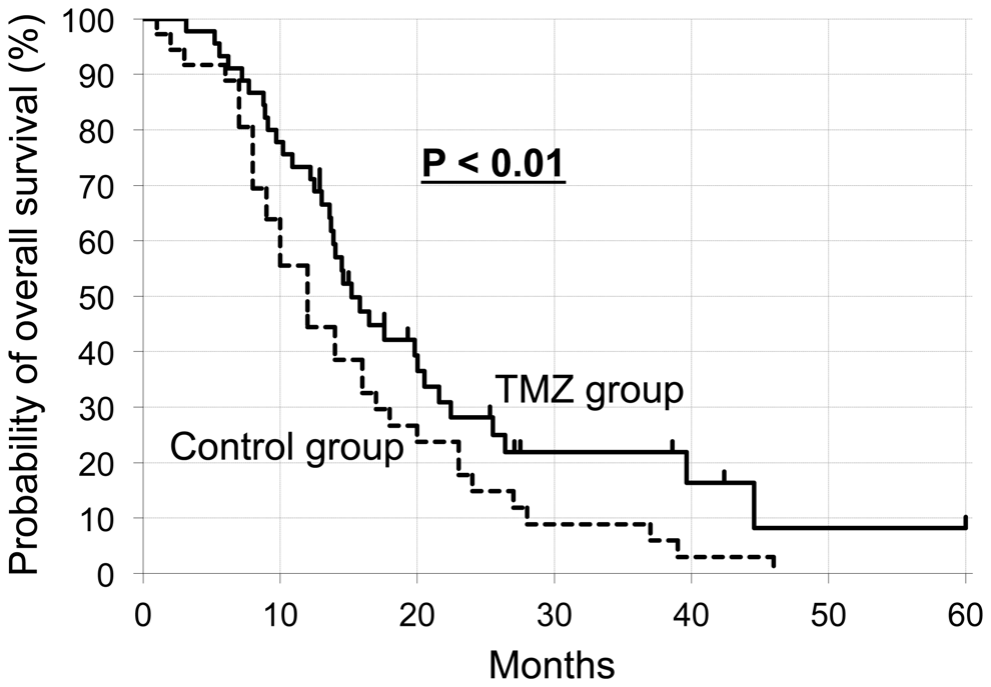
Kaplan-Meier estimates of overall survival. P-values were calculated using a two-sided log-rank test. TMZ, temozolomide.

**Figure 1** and **Table 2** show the survival of the patients. At the time of the analysis, 85.1% of the patients had died after a median follow-up of 14.0 months (range, 1.0 to 70.2 months). Survival was greater in the TMZ group than the control group throughout the follow-up. The hazard ratio for death in the TMZ group relative to the control group was 0.52, with a 95% confidence interval (CI) from 0.27 to 0.77 (P<0.01 by two-sided log-rank test). The median survival was 15.8 months (95% CI, 12.3–19.3 months) in the TMZ group and 12.0 months (95% CI, 9.7–14.3 months) in the control group. Thus, the addition of TMZ resulted in a median survival benefit of 4.0 months. The survival rates of patients in the TMZ group were 71.1% (95% CI, 57.5–84.7%) at 1 year, 27.7% (95% CI, 13.2–42.2%) at 2 years and 21.6% (95% CI, 7.9–35.3%) at 3 years. The survival rates of patients in the control group were 43.8% (95% CI, 26.8–60.8%) at 1 year, 14.6% (95% CI, 2.4–26.8%) at 2 years and 8.8% (95% CI, −1.0–18.6%) at 3 years.

**Table 2:** Kaplan-Meier estimates of overall survival.

	CTR (n = 36)	TMZ (n = 45)
Hazard ratio	1.0	0.52 (0.27–0.77)
Median survival (months)	12.0 (9.7–14.3)	15.8 (12.3–19.3)
Overall survival (%)		
1-year	43.8 (26.8–60.8)	71.1 (57.5–84.7)
2-year	14.6 (2.4–26.8)	27.7 (13.2–42.2)
3-year	8.8 (−1.0–18.6)	21.6 (7.9–35.3)

Values with 95% confidence interval are shown. CTR, control group; TMZ, temozolomide group.

**Table 3** shows the univariate and multivariate analyses for survival in all patients. Univariate analysis showed that extent of surgery and use of TMZ had a significant impact on survival. The extent of surgery had the strongest impact on survival (P<0.01), while the use of TMZ had the second strongest impact on survival (P = 0.049). Multivariate analysis also revealed that the extent of surgery and use of TMZ had a significant impact on survival; again, the extent of surgery had the strongest impact on survival (P<0.01), and the use of TMZ had the second impact on survival (P = 0.035).

**Table 3:** Univariate and multivariate analysis for survival.

Variables	Univariate	Multivariate
	*P-value*	*P-value*
Age		
<60 years vs. ≥60 years	0.45	0.30
Sex		
Male vs. Female	0.46	0.77
Extent of surgery		
GTR+SR vs. PR+biopsy	<0.01	<0.01
Radiation dose (BED; Gy)		
>72 vs. ≤72	0.75	0.18
TMZ therapy		
Yes vs. No	0.049	0.035
IFN-β therapy		
Yes vs. No	0.91	0.69

GTR, gross total removal; SR, subtotal removal; PR, partial removal; TMZ, temozolomide; BED, biological effective dose; IFN-β, interferon-β.

**Figure 2:**
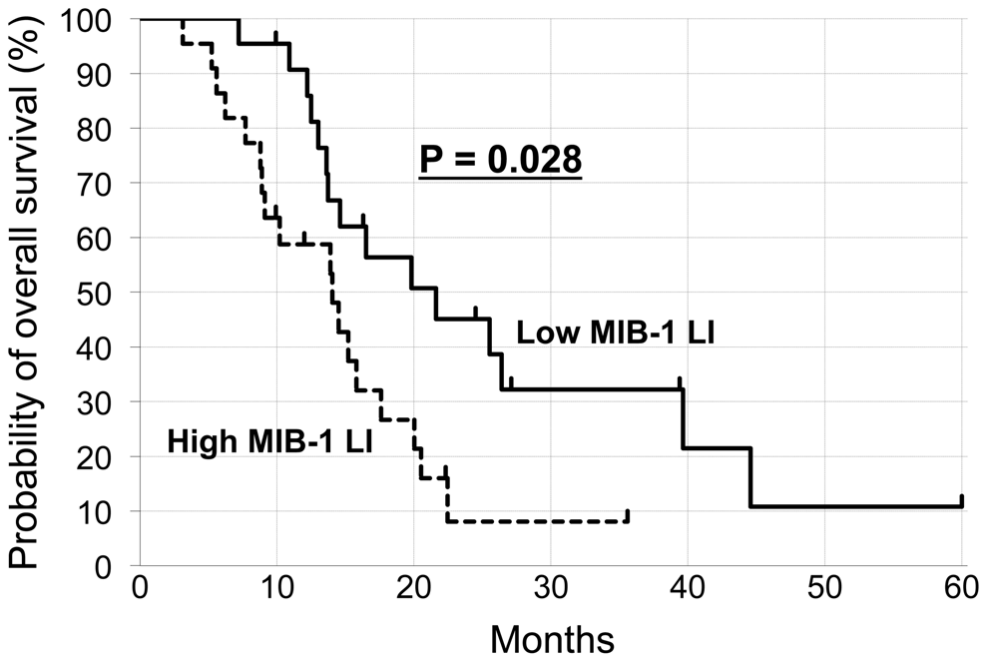
Kaplan-Meier estimates of overall survival in TMZ-treated patients with high and low MIB-1 LI. Cut-off value for MIB-1 LI was set at 23%. P-values were calculated using a two-sided log-rank test.

**Table 4** shows univariate and multivariate analysis for the survival of patients in the TMZ group. Univariate analysis showed that the extent of surgery and MIB-1 LI had significant impacts on survival (P<0.01), while there was no significant correlation between MGMT expression and survival (P = 0.53). Multivariate analysis revealed that MIB-1 LI had a significant impact on survival (P<0.01). As shown in **Figure 2**, patients with low MIB-1 LI (≤ 23%) lived significantly longer than those with high MIB-1 LI (> 23%) (P = 0.028 by two-sided log-rank test).

**Table 4:** Univariate and multivariate analysis for survival in the TMZ group.

Variables	Univariate	Multivariate†	
	*P-value*		*P-value*
Age			
<60 years vs. ≥60 years	0.30		–
Sex			
Male vs. Female	0.83		–
Extent of surgery			
GTR+SR vs. PR+biopsy	<0.01		0.058
Radiation dose (BED; Gy)			
≥72 vs.<72	0.47		–
IFN-β therapy			
Yes vs. No	0.14		0.92
MGMT expression			
Positive vs. Negative	0.53		–
MIB-1 labeling index (%)			
>23 vs. ≤23	<0.01		<0.01

† Potential prognostic factors of which P-value was calculated as <0.15 were evaluated by multivariate analysis using stepwise method. TMZ, temozolomide; GTR, gross total removal; SR, subtotal removal; PR, partial removal; BED, biological effective dose; IFN-β, interferon-β; MGMT, O6-methylguanine DNA metyltransferase.

The incidence and degree of hematologic toxicity observed in the patients in the TMZ group were separately analyzed during the concomitant RT and TMZ periods and the adjuvant TMZ period. During the concomitant RT and TMZ period, grade 3 or 4 leukopenia, neutropenia, thrombocytopenia and anemia evaluated by Common Terminology Criteria for Adverse Events v4.0. was documented in 5 patients (11%), 4 patients (8.8%), 0 patients (0.0%) and 3 patients (6.7%), respectively (**Table 5**). In contrast, no hematologic toxicity of grade 3 or higher was observed during the adjuvant TMZ period. Overall, 9 patients (20.4%) experienced grade 3 or 4 hematologic toxic effects.

**Table 5:** Grade 3 or 4 hematologic toxicity in patients treated with temozolomide.

Toxic effect	Grade 3	Grade 4
	*Number of patient (%)*	*Number of patient (%)*
Leukopenia	3 (6.7)	2 (4.4)
Neutropenia	3 (6.7)	1 (2.2)
Thrombocytopenia	0 (0.0)	0 (0.0)
Anemia	3 (6.7)	0 (0.0)
Any	7 (16.0)	2 (4.4)

Hematologic toxic effects were evaluated by Common Terminology Criteria for Adverse Events v4.0.

## Discussion

The present study showed that glioblastoma patients initially treated with a combined treatment of RT plus TMZ had significantly longer survival than historical controls, with a median survival benefit of 4.0 months. The survival advantage of the combined treatment lasted for at least 3 years. Multivariate analysis showed that TMZ therapy was the second most significant factor influencing survival after the extent of surgery. Although the present study is retrospective and took place in a single institution, to the best of our knowledge, this is the first report investigating the effect of combined therapy with RT and TMZ on Japanese patients with newly diagnosed glioblastoma.

Our results on the survival of TMZ-treated patients were in line with the results of the randomized phase III trial by the EORTC and NCIC (Stupp trial) [6]. However, there were several differences between the two studies that might have affected their clinical outcomes. First, the number of TMZ-treated patients who received GTR was smaller in the present study than in the Stupp trial (15.6% vs. 39.4%). This difference could be a negative bias for survival in the present study because the extent of surgery is one of the most significant prognostic factors in glioblastoma, as most previous studies have described. Second, the incidence of hematologic toxicity of grade 3 or higher was greater in the present study than in the Stupp trial during the concomitant RT and TMZ periods (20.4% vs. 6.7%). Third, adjuvant TMZ was continued as long as it was tolerable in the present study, while a maximum of 6 cycles were administered in the Stupp trial. Interestingly, in contrast with the concomitant period, the incidence of hematologic toxicity of grade 3 or higher was lower in the present study than the Stupp trial (0.0% vs. 14.3%) during the adjuvant period. As a result, the average number of adjuvant TMZ cycles administered in the present study was 7. The number of patients completing 6 cycles of TMZ treatment was greater in our study than the Stupp trial (57.7% vs. 47.1%). It is difficult to conclude how all of the differences described above actually affected the clinical outcome. However, the results of the present study indicate that the addition of TMZ to RT is beneficial for the survival for Japanese patients with newly diagnosed glioblastoma.

There are several possible reasons for the more severe hematologic toxicity observed in the concomitant RT and TMZ period. (i) We treated nearly all (95.7%) of the patients with newly diagnosed and histologically confirmed glioblastoma using TMZ. Therefore, there is a possibility that our cohort included a greater number of patients with unfavorable general conditions than the Stupp trial. (ii) The dose of TMZ used in the Stupp trial may be excessive for Japanese patients. (iii) Japanese individuals may be more susceptible to the concomitant use of TMZ with RT than others because of racial differences in single nucleotide polymorphisms. Further investigations are needed.

MGMT status is recognized as a strong predictor for the survival of newly diagnosed glioblastoma patients treated with RT and TMZ. MGMT is involved in the repair of DNA damage through the removal of the alkyl unit added by TMZ from the O^6^ position of desoxy-guanine [10]. Thus, in theory, the functional loss of MGMT enhances the cytotoxicity of TMZ. Previous studies have demonstrated that the methylation of the *MGMT* promoter, which leads to the silencing of *MGMT* transcription, has a significant impact on the response to TMZ-based therapy and the survival in patients with newly diagnosed glioblastoma [6], [11], [12]. In most of the previous studies, the methylation status of the *MGMT* promoter has been evaluated by with methylation-specific polymerase chain reaction (MS-PCR). Meanwhile, Quillien *et al*. compared the predictive values of 5 techniques for assessing MGMT status in a series of 100 newly diagnosed glioblastoma patients treated with the Stupp protocol [8]. In their study, 5 techniques, MS-PCR, pyrosequencing, immunohistochemistry, methylation-sensitive high-resolution melting and high-throughput quantitative methylation assay (MethyLight), were investigated. As a result, pyrosequencing, MS-PCR and immunohistochemistry had significant predictive value for overall survival. In the present study, we selected immunohistochemical method to assess MGMT expression status because the amount of pathological samples was insufficient for performing MS-PCR. However, our results showed no significant correlation between MGMT expression and survival, even though the evaluation criteria used were the same as those of Quillien’s study. In other previous studies, the correlation between immunohistochemically determined MGMT expression and the survival of glioblastoma patients treated with TMZ is controversial [8], [13]–[15]. Taken together, the results of the current study suggest that immunohistochemistry analysis may not be suitable for assessing MGMT expression status as a predictor for the survival in such cases. Further investigation is warranted.

MIB-1, an antibody reactive to Ki-67 protein, is involved in cell-cycle progression. MIB-1 LI has been widely used as a cell proliferation marker in a variety of cancers [16], [17]. Previous studies have shown that MIB-1 LI is correlated with poorer survival in gliomas across all histological grades [18]. However, the predictive value of MIB-1 LI for the survival of glioblastoma patients treated with RT and TMZ has not been well established. In the present study, MIB-1 LI with a cutoff at the median value of 23% was shown to be a significant predictor of survival by both univariate and multivariate analyses. Moreover, significantly shorter survival of TMZ-treated patients with high MIB-1 LI was shown by Kaplan-Meier estimates (**Fig. 2**). These data may point toward a strategy of increasing treatment intensity in patients with high MIB-1 LI. Taken together, further investigation employing larger cohorts will aid the exploration of useful predictors for survival, including MGMT status assessed by various methods and MIB-1 LI, in patients with glioblastoma treated with the combined RT and TMZ.

The current study and others have demonstrated that combined treatment with RT and TMZ significantly improves the survival of patients with newly diagnosed glioblastoma compared with historical controls [6], [19]. However, the results also clearly show that this combined therapy is not curative. Thus, further development of novel treatment strategies for glioblastoma is required. The addition of emerging molecular-targeted drugs to combined RT and TMZ has been tested. Thus far, the addition of erlotinib (EGFR tyrosine kinase inhibitor) and bevacizumab (humanized monoclonal antibody against vascular endothelial growth factor) has resulted in a measure of therapeutic gain [20], [21]. Another potential direction that has generated several promising results is dose escalation in RT using intensity-modulated radiation therapy (IMRT) techniques [22], [23]. Our institute has been treating glioblastoma patients with a regimen consisting of 3DCRT (2 Gy/fraction for the first 46 Gy) and the following IMRT (3 Gy/fraction to GTV and 2 Gy/fraction to the area with 1.5-cm margin surrounding GTV for total of 7 fractions), along with TMZ, since January 2012. Further investigation to explore the survival benefit from these novel treatment strategies, including our dose escalation study, is needed.

## References

[1] Suzuki Y, Shirai K, Oka K, Mobaraki A, Yoshida Y, et al (2010) Higher pAkt expression predicts a significant worse prognosis in glioblastomas. J Radiat Res 51: 343–348. 20410674 10.1269/jrr.09109

[2] Louis DN, Ohgaki H, Weistler OD, Cavenee WK, Burger PC, et al (2007) The 2007 WHO classification of tumours of the central nervous system. Acta Neuropathol 114: 97–109. 17618441 10.1007/s00401-007-0243-4 PMC1929165

[3] Walker MD, Green SB, Byar DP, Alexander E Jr, Batzdorf U, et al (1980) Randomized comparisons of radiotherapy and nitrosoureas for the treatment of malignant glioma after surgery. N Engl J Med 303: 1323–1329. 7001230 10.1056/NEJM198012043032303

[4] Stewart LA (2002) Chemotherapy in adult high-grade glioma: a systematic review and meta-analysis of individual patient data from 12 randomised trials. Lancet 359: 1011–1018. 11937180 10.1016/s0140-6736(02)08091-1

[5] Stupp R, Mason WP, van den Bent MJ, Weller M, Fisher B, et al (2005) Radiotherapy plus concomitant and adjuvant temozolomide for glioblastoma. N Engl J Med 352: 987–996. 15758009 10.1056/NEJMoa043330

[6] Stupp R, Hegi ME, Mason WP, van den Bent MJ, Taphoorn MJ, et al (2009) Effects of radiotherapy with concomitant and adjuvant temozolomide versus radiotherapy alone on survival in glioblastoma in a randomised phase III study: 5-year analysis of the EORTC-NCIC trial. Lancet Oncol 10: 459–466. 19269895 10.1016/S1470-2045(09)70025-7

[7] Nagaishi M, Yokoo H, Hirato J, Yoshimoto Y, Nakazato Y (2012) Clinico-pathological feature of pilomyxoid astrocytomas: three case reports. Neuropathology 31: 152–157. 10.1111/j.1440-1789.2010.01143.x 20667008

[8] Quillien V, Lavenu A, Karayan-Tapon L, Carpentier C, Labussiere M, et al (2012) Comparative assessment of 5 methods (methylation-specific polymerase chain reaction, MethyLight, pyrosequencing, methylation-sensitive high-resolution melting, and immunohistochemistry) to analyze O^6^-methylguanine-DNA- methyltranferase in a series of 100 glioblastoma patients. Cancer 118: 4201–4211. 22294349 10.1002/cncr.27392

[9] Tanaka G, Nakazato Y (2004) Automatic quantification of the MIB-1 immunoreactivity in brain tumors. Int Congr Ser 1259: 15–19.

[10] Gerson SL (2004) MGMT: its role in cancer aetiology and cancer therapeutics. Nat Rev Cancer 4: 296–307. 15057289 10.1038/nrc1319

[11] Hegi ME, Diserens AC, Gorlia T, Hamou MF, de Tribolet N, et al (2005) MGMT gene silencing and benefit from temozolomide in glioblastoma. N Engl J Med 352: 997–1003. 15758010 10.1056/NEJMoa043331

[12] Weller M, Felsberg J, Hartmann C, Berger H, Steinbach JP, et al (2009) Molecular predictors of progression-free and overall survival in patients with newly diagnosed glioblastoma: a prospective translational study of the German Glioma Network. J Clin Oncol 27: 5743–5750. 19805672 10.1200/JCO.2009.23.0805

[13] Chinot OL, Barrie M, Fuentes S, Eudes N, Lancelot S, et al (2007) Correlation between O^6^-methylguanine-DNA methyltransferase and survival in inoperable newly diagnosed glioblastoma patients treated with neoadjuvant temozolomide. J Clin Oncol 25: 1470–1475. 17442989 10.1200/JCO.2006.07.4807

[14] Preusser M, Janzer RC, Felsberg J, Reifenberger G, Hamou MF, et al (2008) Anti-O^6^-methylguanine-methyltransferase (MGMT) immunohistochemistry in glioblastoma multiforme: observer variability and lack of association with patient survival impede its use as clinical biomarker. Brain Pathol 18: 520–532. 18400046 10.1111/j.1750-3639.2008.00153.x PMC8095504

[15] Tang K, Jin Q, Yan W, Zhang W, You G, et al (2012) Clinical correlation of MGMT protein expression and promoter methylation in Chinese glioblastoma patients. Med Oncol 29: 1292–1296. 21394635 10.1007/s12032-011-9901-4

[16] Brown DC, Gatter KC (2002) Ki67 protein: the immaculate deception? Histopathology 40: 2–11. 11903593 10.1046/j.1365-2559.2002.01343.x

[17] Colleoni M, Zarieh D, Gelber RD, Viale G, Luini A, et al (2003) Preoperative systemic treatment: prediction of responsiveness. Breast 12: 538–542. 14659132 10.1016/s0960-9776(03)00163-2

[18] Cunningham JM, Kimmel DW, Scheithauer BW, O’Fallon JR, Novotny PJ, et al (1997) Analysis of proliferation markers and p53 expression in gliomas of astrocytic origin: relationships and prognostic value. J Neurosurg 86: 121–130. 8988090 10.3171/jns.1997.86.1.0121

[19] Athanassiou H, Synodinou M, Maragoudakis E, Paraskevaidis M, Verigos C, et al (2005) Randomized phase II study of temozolomide and radiotherapy compared with radiotherapy alone in newly diagnosed glioblastoma multiforme. J Clin Oncol 23: 2372–2377. 15800329 10.1200/JCO.2005.00.331

[20] Prados MD, Chang SM, Butowski N, DeBoer R, Parvataneni R, et al (2009) Phase II study of erlotinib plus temozolomide during and after radiation therapy in patients with newly diagnosed glioblastoma multiforme or gliosarcoma. J Clin Oncol 27: 579–584. 19075262 10.1200/JCO.2008.18.9639 PMC2645859

[21] Lai A, Tran A, Nghiemphu PL, Pope WB, Solis OE, et al (2011) Phase II study of bevacizumab plus temozolomide during and after radiation therapy for patients with newly diagnosed glioblastoma multiforme. J Clin Oncol 29: 142–148. 21135282 10.1200/JCO.2010.30.2729 PMC3058273

[22] Tsien CI, Brown D, Normolle D, Schipper M, Piert M, et al (2012) Concurrent temozolomide and dose-escalated intensity-modulated radiation therapy in newly diagnosed glioblastoma. Clin Cancer Res 18: 273–279. 22065084 10.1158/1078-0432.CCR-11-2073 PMC3266840

[23] Iuchi T, Hatano K, Narita Y, Kodama T, Yamaki T, et al (2006) Hypofractionated high-dose irradiation for the treatment of malignant astrocytomas using simultaneous integrated boost technique by IMRT. Int J Radiat Oncol Biol Phys 64: 1317–1324. 16580493 10.1016/j.ijrobp.2005.12.005

